# Artificial Intelligence and Digital Biomarkers in Hepatology: Critical Perspectives, Emerging Evidence, and Future Directions

**DOI:** 10.7759/cureus.92639

**Published:** 2025-09-18

**Authors:** Pulkit Mehrotra, Vengadakrishnan K, Punit Mehrotra

**Affiliations:** 1 Medicine, Sri Ramachandra Institute of Higher Education and Research, Chennai, IND; 2 General Medicine, Sri Ramachandra Medical College and Research Institute, Chennai, IND; 3 Gastroenterology and Hepatology, Lucknow Gastroenterology and Gynaecology Center, Lucknow, IND

**Keywords:** artificial intelligence, cirrhosis, digital biomarkers, hepatocellular carcinoma, liver disease, machine learning, predictive analytics, wearable technology

## Abstract

Liver diseases are a major global health burden, responsible for nearly two million deaths worldwide each year. Despite advances in imaging, serology, and non-invasive fibrosis assessment, late-stage diagnosis persists, limiting curative interventions. Artificial intelligence (AI) and digital biomarkers promise to transform hepatology by enhancing early detection, risk stratification, and remote monitoring. This review provides a critical synthesis of recent evidence in AI-driven imaging, digital histopathology, predictive modeling using electronic health records (EHR), and wearable-based phenotyping. We compare and analyze the strengths and limitations of landmark AI models, highlight real-world implementation barriers such as algorithmic bias and data privacy, and explore emerging paradigms such as federated learning and multimodal integration. While AI tools consistently outperform conventional scores (e.g., Model for End-Stage Liver Disease [MELD]) in predictive accuracy, their clinical adoption remains limited by regulatory, ethical, and validation challenges. In the future, hepatology will require equitable AI systems trained on diverse datasets, integration into electronic medical record (EMR) workflows, and patient-centered digital health platforms. Establishing global AI liver disease registries and multicenter validation trials will be critical to ensure equitable and scalable clinical adoption.

## Introduction and background

Liver disease is a leading cause of mortality, with cirrhosis and hepatocellular carcinoma (HCC) responsible for over two million deaths annually, accounting for approximately 4% of global deaths [1]. Despite advances in imaging and serology, most cases are detected at advanced stages, limiting curative options and contributing to preventable mortality, particularly in resource-limited settings. Current diagnostic modalities - biopsy, imaging, elastography, and clinical scoring systems such as Model for End-Stage Liver Disease (MELD) and Child-Pugh -are limited by invasiveness, delayed detection, and interobserver variability [2,3]. In recent years, Artificial intelligence (AI)-driven analytics and digital biomarkers have emerged as practical tools for earlier detection and risk prediction. AI refers to computer programs that learn patterns from data; deep learning (e.g., convolutional neural networks [CNNs]) is used for images, while other machine-learning methods analyze numbers and text. By combining multiple sources - medical imaging, electronic health records (EHRs), laboratory biomarkers, and wearable-device signals - AI can reveal hidden disease patterns that are often invisible to traditional assessment [4,5]. However, much of the existing literature focuses on single-modality studies without large-scale external validation, and many reviews summarize applications without comparing performance, feasibility, or bias. This review brings these strands together by synthesizing diverse AI modalities in hepatology (imaging, pathology, EHR, wearables), critically analyzing advantages and limitations, and highlighting future research priorities for equitable and explainable AI in liver disease.

## Review

This review involved a narrative synthesis of literature. A structured search of PubMed, Scopus, and Web of Science databases was performed for English-language publications between 2018 and 2025, using combinations of the terms: “artificial intelligence,” “machine learning,” “deep learning,” “digital biomarkers,” “hepatology,” “liver disease,” “fibrosis,” and “hepatocellular carcinoma.” Inclusion criteria were studies reporting AI or digital biomarker applications in hepatology with clinical, imaging, histopathological, or wearable data. Opinion articles without empirical evidence were excluded. Reference lists of relevant articles were manually screened to identify additional studies. As this is a narrative rather than a systematic review, risk-of-bias assessments were not performed, but methodological quality and limitations of key studies are critically discussed.

AI in liver imaging

Beyond Proof-of-Concept: Advances in Imaging-Based AI

**Figure 1:**
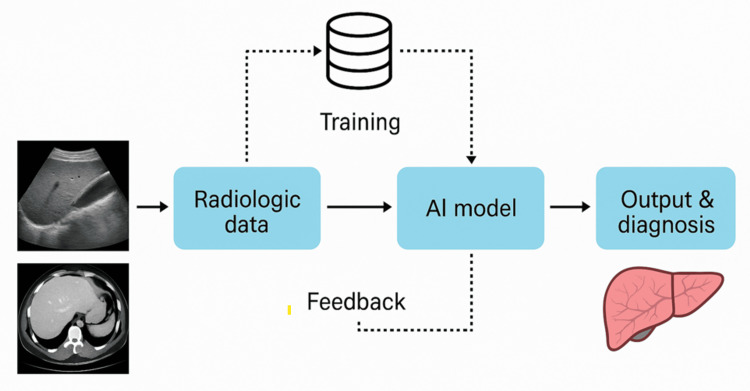
Workflow of AI in liver imaging Radiologic data (ultrasound, CT, MRI, and elastography) are input into deep learning models for fibrosis staging, cirrhosis detection, and HCC prediction. Outputs integrate with EMR decision support Original figure created for this manuscript. No external copyrighted material was used AI: artificial intelligence; CT: computed tomography; MRI: magnetic resonance imaging; HCC: hepatocellular carcinoma; EMR: electronic medical record

Recent deep-learning models have achieved an area under the receiver operating characteristic curve (AUC) >0.90 for staging liver fibrosis using ultrasound/shear-wave elastography [6-8]. (AUC summarizes overall accuracy across thresholds: 0.5 = no better than chance; 1.0 = perfect.) MRI-based models integrating multiparametric sequences further improved diagnostic sensitivity for cirrhosis detection [9]. Figure 1 illustrates the typical AI-assisted liver imaging workflow, from acquisition of ultrasound, CT, and MRI data to deep learning model interpretation and fibrosis staging outputs.

However, critical analysis reveals three persistent gaps:

Limited Generalizability

Most models are trained on single-center datasets with homogeneous populations, leading to reduced accuracy in underrepresented cohorts [10].

Clinical Utility vs. Complexity

High-performance models often require computational infrastructure unavailable in low-resource settings, limiting their translation into routine hepatology practice.

Explainability

Despite high accuracy, CNNs often operate as “black boxes,” making it difficult for radiologists to interpret why a model predicts advanced fibrosis in a given case [11].

Comparative Evidence

**Figure 2:**
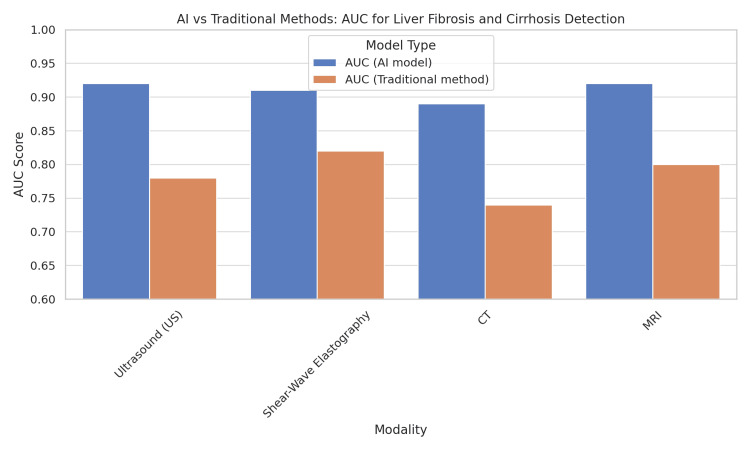
Comparison of AI versus traditional diagnostic accuracy for liver fibrosis and cirrhosis Bar chart showing mean AUC ± SD for ≥F2 fibrosis detection: transient elastography (AUC 0.72), MRI (0.78), AI-enhanced elastography (0.89), and multiparametric MRI-AI models (0.92). P<0.05 is considered significant Original figure created for this manuscript. No external copyrighted material was used AI: artificial intelligence; CT: computed tomography; MRI: magnetic resonance imaging; AUC: area under the receiver operating characteristic curve; SD: standard deviation

A recent multicenter meta-analysis by Smith et al. (2024) showed that AI-enhanced MRI outperformed transient elastography (TE) with a sensitivity of 88% vs. 72% for ≥F3 fibrosis, but also noted decreased specificity in fatty liver disease populations [12]. This highlights AI’s diagnostic advantage but also underscores the need for broader validation in diverse liver disease populations. Figure 2 provides a comparison of the diagnostic performance (AUC values) of AI-enhanced imaging versus conventional approaches for fibrosis and cirrhosis detection, showing a consistent 10-20% performance gain.

Key takeaway: While AI imaging outperforms conventional radiology, robust external validation and cost-benefit analyses are essential before widespread clinical integration.

Digital histopathology and virtual biopsy

Current Promise, Future Potential

Digital pathology with AI-assisted scoring enables continuous fibrosis quantification, reducing interobserver variability [13]. For nonalcoholic steatohepatitis (NASH), deep learning models have demonstrated >90% concordance with expert hepatopathologists in grading steatosis, ballooning, and inflammation [14,15]. However, virtual biopsy - predicting histology entirely from non-invasive imaging - remains an emerging field. Recent studies integrating MRI-proton density fat fraction (MRI-PDFF) with deep learning have shown potential to replace biopsy in select NASH trials [16]. However, no AI histopathology models have yet received regulatory approval for routine NASH drug trials, reflecting the gap between research and clinical implementation. Yet critical challenges remain: histology is still required for drug development endpoints in NASH. AI models may not capture rare histologic patterns seen in autoimmune or cholestatic liver diseases. Lack of standardized digital pathology pipelines hinders model reproducibility across centers.

Key takeaway: Digital histopathology enhances scoring reproducibility but cannot yet fully replace biopsy in all hepatology contexts.

Predictive modeling using EHR and multimodal data

Machine-learning models applied to EHRs can outperform traditional scores such as MELD and the Child-Pugh. For example, Kim et al. trained a gradient-boosting model using routine labs and clinical variables to predict 30-day mortality in decompensated cirrhosis (AUC 0.84) vs. MELD (AUC 0.71) [17]. Cheung et al. developed an EHR-based risk model for HCC in chronic hepatitis B (HBV) with an AUC of 0.85 vs. 0.70 for conventional scores [18]. Natural-language processing (NLP) applied to clinical notes has also identified phenotypic markers of undiagnosed cirrhosis, improving early detection rates by 32% [19]. While promising, EHR-based AI models require harmonized multicenter datasets to overcome institutional data silos and ensure generalizability.

Critical insights

Data fragmentation across institutions limits the training of robust multimodal models, and most EHR-based AI lacks prospective validation in diverse healthcare settings. Integration into clinician-friendly EHR dashboards remains a barrier. Wearables and digital phenotyping: home-based heart-rate variability (HRV) metrics from smartwatches predicted 90-day mortality [20]; sweat biosensors measuring cytokines (IL-6, TNF-α) detected early inflammation preceding clinical decompensation [21]; and smartphone speech analytics detected minimal hepatic encephalopathy [22]. Across these domains, AI improves accuracy and monitoring but requires careful validation and attention to bias before broad adoption. To synthesize findings from imaging, pathology, EHR models, and wearables, Table 1 compares key AI models with conventional hepatology tools. [Table 1] summarizes key studies comparing AI models with conventional hepatology tools, highlighting their relative performance and limitations.

**Table 1:** Comparative performance of AI models versus conventional hepatology tools Data expressed as mean AUC ± SD where available. P<0.05 is considered statistically significant. AI consistently improves sensitivity and AUC vs. conventional approaches, but real-world integration remains limited AI: artificial intelligence; HCC: hepatocellular carcinoma; TE: Transient elastography; MRI: magnetic resonance imaging; MELD: Model for End-Stage Liver Disease; CNN: convolutional neural network; ML: machine learning; HBV: hepatitis B virus; EHR: electronic health record; AUC: area under the receiver operating characteristic curve; SD: standard deviation

Domain	Conventional tool	AI model	Key performance metrics	Limitations
Fibrosis staging	TE	CNN on shear-wave elastography [10]	AUC 0.72 (TE) vs. AUC 0.92 (AI) for ≥F3	Needs high-quality elastography datasets
Cirrhosis detection	MRI + radiologist report	Multiparametric MRI + deep learning [11]	Sensitivity 72% (MRI) vs. 88% (AI)	External validation limited
HCC risk	PAGE-B, REACH-B scores	ML models on HBV cohort [18]	AUC 0.70 (scores) vs. 0.85 (AI model)	Bias in minority populations
Cirrhosis mortality	MELD, Child-Pugh	Random forest on EHR data [17]	AUC 0.71 (MELD) vs. 0.84 (AI) for 30-day mortality	Data fragmentation across hospitals
Encephalopathy	Psychometric testing	Smartphone speech analytics [22]	Correlation 0.65 (tests) vs. 0.82 (AI)	Limited linkage to clinical outcomes

Ethical considerations and barriers to adoption

Algorithmic Bias

Studies show lower AI model accuracy in women and ethnic minorities due to underrepresentation in training datasets [23]. For example, an AI liver fibrosis model trained on predominantly male viral hepatitis cohorts showed reduced accuracy in female patients with NASH, highlighting population bias concerns.

Explainability and Trust

Lack of interpretability hinders clinician confidence and regulatory approval. Emerging explainability tools like SHAP and LIME may help clinicians understand AI decision pathways, improving trust and adoption.

Privacy and Security

Wearable and cloud-integrated systems must comply with strict HIPAA/GDPR data regulations [24]. Similarly, a 2025 multicenter prospective validation demonstrated deep learning models achieving high sensitivity for early hepatocellular carcinoma detection, further highlighting AI’s potential for liver cancer surveillance [25]. Recent international studies have successfully used federated learning to harmonize liver fibrosis staging models across diverse cohorts without compromising data privacy [26].

Emerging solutions include: Federated learning to train AI models without centralizing patient data; Explainable AI (XAI) frameworks for interpretable decision-making; Independent regulatory sandboxes to test AI tools under controlled conditions before scaling.

Future research priorities

Multimodal Integration

Combining imaging, lab biomarkers, genomics, and wearables for holistic disease profiling. Developing prospective randomized trials to test AI-assisted surveillance pathways against standard hepatology care is essential to establish real-world benefit.

Federated Data Collaboratives

Global consortia ensuring diverse, representative datasets for equitable AI.

Prospective Clinical Trials

Evaluating AI-assisted care pathways vs. standard hepatology practice for real-world impact. Explainable and Patient-Centered AI: Co-developing models with patient advocates and hepatologists for transparent deployment.

## Conclusions

AI and digital biomarkers hold great promise for earlier detection and improved management of liver diseases. However, they are complementary to, not replacements for, conventional scoring systems at present. For successful clinical adoption, external validation, equitable datasets, and integration into EMR workflows are essential. The future of the field of hepatology will depend on collaborative efforts between clinicians, data scientists, regulators, and patients to ensure safe, ethical, and meaningful use of AI.
